# A New Multidimensional Repulsive Potential Field to Avoid Obstacles by Nonholonomic UAVs in Dynamic Environments

**DOI:** 10.3390/s21227495

**Published:** 2021-11-11

**Authors:** Cezary Kownacki, Leszek Ambroziak

**Affiliations:** Department of Robotics and Mechatronics, Faculty of Mechanical Engineering, Bialystok University of Technology, Wiejska St. 45C, 15-351 Bialystok, Poland; l.ambroziak@pb.edu.pl

**Keywords:** fixed-wing UAV, nonholonomic UAV, repulsive potential field, obstacle avoidance, dynamical environments

## Abstract

The ability of autonomous flight with obstacle avoidance should be a fundamental feature of all modern unmanned aerial vehicles (UAVs). The complexity and difficulty of such a task, however, significantly increase in cases combining moving obstacles and nonholonomic UAVs. Additionally, since they assume the symmetrical distribution of repulsive forces around obstacles, traditional repulsive potential fields are not well suited for nonholonomic vehicles. The limited maneuverability of these types of UAVs, including fixed-wing aircraft, requires consideration not only of their relative position, but also their speed as well as the direction in which the obstacles are moving. To address this issue, the following work presents a novel multidimensional repulsive potential field dedicated to nonholonomic UAVs. This field generates forces that repulse the UAV not from the obstacle’s geometrical center, but from areas immediately behind and in front of it located along a line defined by the obstacle’s velocity vector. The strength of the repulsive force depends on the UAV’s distance to the line representing the obstacle’s movement direction, distance to the obstacle along that line, and the relative speed between the UAV and the obstacle projected to the line, making the proposed repulsive potential field multidimensional. Numerical simulations presented within the paper prove the effectiveness of the proposed novel repulsive potential field in controlling the flight of nonholonomic UAVs.

## 1. Introduction

Within the last decade, the field of Unmanned Aerial Systems (UAS) has been experiencing a true revolution and is now used in both military and civilian applications. The range of these applications can further be increased through a fully functional obstacle avoidance system [1,2]. Although the creation of such systems that are both effective and reliable is still extremely challenging [3], it is necessary to ensure an appropriate level of security during missions that are carried out in various situations [4,5]. Over the years numerous obstacle avoidance mechanisms for mobile robots have been developed. These can be divided into one-step and multi-step methods. The former directly reduce the sensor information to motion control. This group of methods includes heuristic ([6]) methods and physical analogies methods. Methods of physical analogies liken obstacle avoidance to a known physical problem. One category of these types of systems is artificial potential field methods or PFM’s [7] which, to put it simply, use an analogy in which the robot is treated as a particle that moves within a space through the influence of various force fields. Its target location exerts a force that attracts the particle while obstacles exert repulsive forces. There exist many methods of obstacle avoidance that are based on potential field methods. The traditional artificial potential field method is computationally simple, easy to implement, and, within a static environment, effective in avoiding obstacles. However, despite all these advantages, there are also numerous problems, including, for example, unreachable targets, the formation of local minima, and poor effectiveness in dynamic environments. In their work [8], Park et al. used a simulated annealing algorithm that displayed good stability to optimize the path of a robot so that it could reach its target quickly. He et al., on the other hand, proposed a good quality algorithm containing a module that allowed escaping local minima to obtain the global optimal solution iteratively ensuring that the robot reaches its target [9]. The problem of local minima and GNRON (goal non-reachable with obstacles nearby) was solved in an interesting way in work [10]. To resolve the local minima and GNRON problem, this work proposes an intelligent cooperative collision avoidance approach combining the enhanced potential field with a fuzzy inference system. What is important, is that the presented algorithm provides a near-optimal collision-free trajectory. Furthermore, an optimal and collision-free trajectory was proposed in work [11], where an APF approach, called an “enhanced curl-free vector field” was described. In this method, for the repulsive potential field, one computes each angle between the velocity vectors of UAVs and the relative position vectors of moving obstacles to the UAVs. The comparisons of the computed angles and the velocity of UAVs determine the direction of the curl-free vector field. The method, presented in [12], uses the vector superposition method to improve the repulsion field model providing the robot with good obstacle avoidance and the ability to quickly find its target point. Yet another method described in [13] proposes improvements to the traditional artificial potential field method through increasing the safety distance of the repulsion potential field. Most of the methods described above are generally adapted for static environments and do not take into account moving obstacles. Ge and Cui [14], however, were successful in improving some artificial potential field obstacle avoidance techniques and did consider obstacle dynamics. In their work, Ruiz et al. [15] propose a real-time collision-free path-planning algorithm for a quadrotor UAV using only onboard visual and inertial sensors. Their solution uses a modified potential field method to overcome the non-reachable goal problem and involves three key components: pattern-based ground texturing for localization, the described above potential field method for path planning, and PD controllers for steering. The method allows the vehicle to avoid known/unknown obstacles and reach the target in a complex indoor environment. Most potential field algorithms are based on the distance between the vehicle and target points within a one-time frame. However, in cases involving moving obstacles, it is also desirable to consider the relative direction of motion as well. An interesting algorithm described in [16] utilizes the cost function for a potential field as the function of the obstacle’s distance and direction. Methods utilizing potential fields for obstacle avoidance are often used to control the formation flight of UAVs and ensure safety and reliability during such operations. In [17], Wen et al. apply a leader–follower formation approach coupled with a potential field method for formation control and obstacle avoidance. However, their solution does not take into account a local minimum of the design potential function within a complex environment. This problem is addressed by [18], where its authors present a modified artificial potential field approach in combination with a goal technique and a virtual structure method to avoid cavities, providing the agent with the possibility to move away from the local minimum in an environment with both static and dynamic obstacles. Another paper [19] resolves local minima and oscillation problems in potential field functions with an unconventional rotating potential field around obstacles. Yet another article [20] presents a virtual leader approach combined with an extended local potential field. This method was designed for small unmanned helicopters and is suitable only for holonomic mobile objects, strongly limiting its application. Obstacle avoidance techniques based on potential fields are, as a rule, more popular for holonomic objects, which may be caused by the fact that methods for nonholonomic flying robots are more involved because of kinematic limitations and the need for the UAVs to constantly remain in motion (no possibility of hovering). An interesting approach to the problem of obstacle avoidance by fixed-wing UAVs has been presented in [21], where the authors use an algorithm that utilizes a morphing potential field for obstacle avoidance. The proposed potential field has a Gaussian shape and uses the norm of distance between the agent and another agent or obstacle with the origin of the field localized at the centroid of the agent/obstacle to be avoided. What is more, the additional reference shifting term has also been included in the distance norm as a means of further shaping the potential to avoid unnecessary levels of cost beyond the avoided obstacle by shifting the potential function origin away from the centroid of the object. A fixed-wing UAV, provided only with the position and velocity of the obstacle, was able to successfully and autonomously depart from its predefined trajectory and avoid a collision with a static obstacle. This approach, however, possesses a big disadvantage, namely, the need to precisely define the parameters of the generated field, which is not an easy task.

The present work also addresses the problem of UAV obstacle avoidance in dynamic environments by using a potential field method that considers the direction of an obstacle’s motion as well as precisely describes an original multidimensional repulsive potential field generation algorithm. The proposed solution for the avoidance of non-stationary obstacles is especially dedicated to non-holonomic UAVs. The potential field generates forces that repulse the UAV not from the geometrical center of the obstacle, but rather from areas behind it and in the front of it located along its velocity vector or its direction of movement. The strength of the repulsive force depends on the distance to the line of the obstacle’s movement direction, the distance to the obstacle along that line, and the relative speed between the UAV and the obstacle projected to the line. The proposed repulsive potential field can, therefore, be treated as a multidimensional potential field. Due to the asymmetrical shape of the area of the designed repulsive field around the moving obstacle, it can be adjusted separately for frontal, rear, and lateral collision scenarios. Thus, the main innovation and contribution of the work concerning spherical repulsive potential fields are the different effects and ranges of repulsion in lateral and perpendicular directions of the obstacle’s movement attained through this solution. The properties of the above-described multidimensional field can be used to precisely adapt repulsion areas around obstacles to the maneuverability of non-holonomic UAVs. The synthesis of the potential field algorithm is precisely described and presented in great detail. The proposed obstacle avoidance system was thoroughly tested and verified during numerical simulations in scenarios that confirm the behavior of the repulsive field in lateral and perpendicular directions, representing two independent extreme cases, to the obstacle’s velocity vector. Positions of the UAV and those of moving (dynamic) obstacles acquired during tests were recorded and then presented on time plots. The obtained results validate the effectiveness of the proposed potential field-based obstacle avoidance method which provides a simple and computationally efficient solution to ensure a collision-free flight of non-holonomic UAVs. An important advantage of the proposed solution is that there is no need to tune algorithm parameters to the dynamics of the UAV such as its minimum turning radius, maneuverability, etc., which was necessary for previously mentioned algorithms.

The remainder of the paper is organized in the following manner: Section 2 includes a thorough description of the proposed multidimensional repulsive potential field and the definitions of input signals (desired heading, pitch angle, and speed) for low-level control loops. Numerical simulations of collision scenarios are described and discussed in Section 3. Section 4 presents the validation of the proposed navigation and obstacle avoidance strategy conducted using computer simulations and illustrative examples. Comprehensive conclusions are presented in Section 5.

## 2. Design of the Multidimensional Repulsive Potential Field

**Figure 1:**
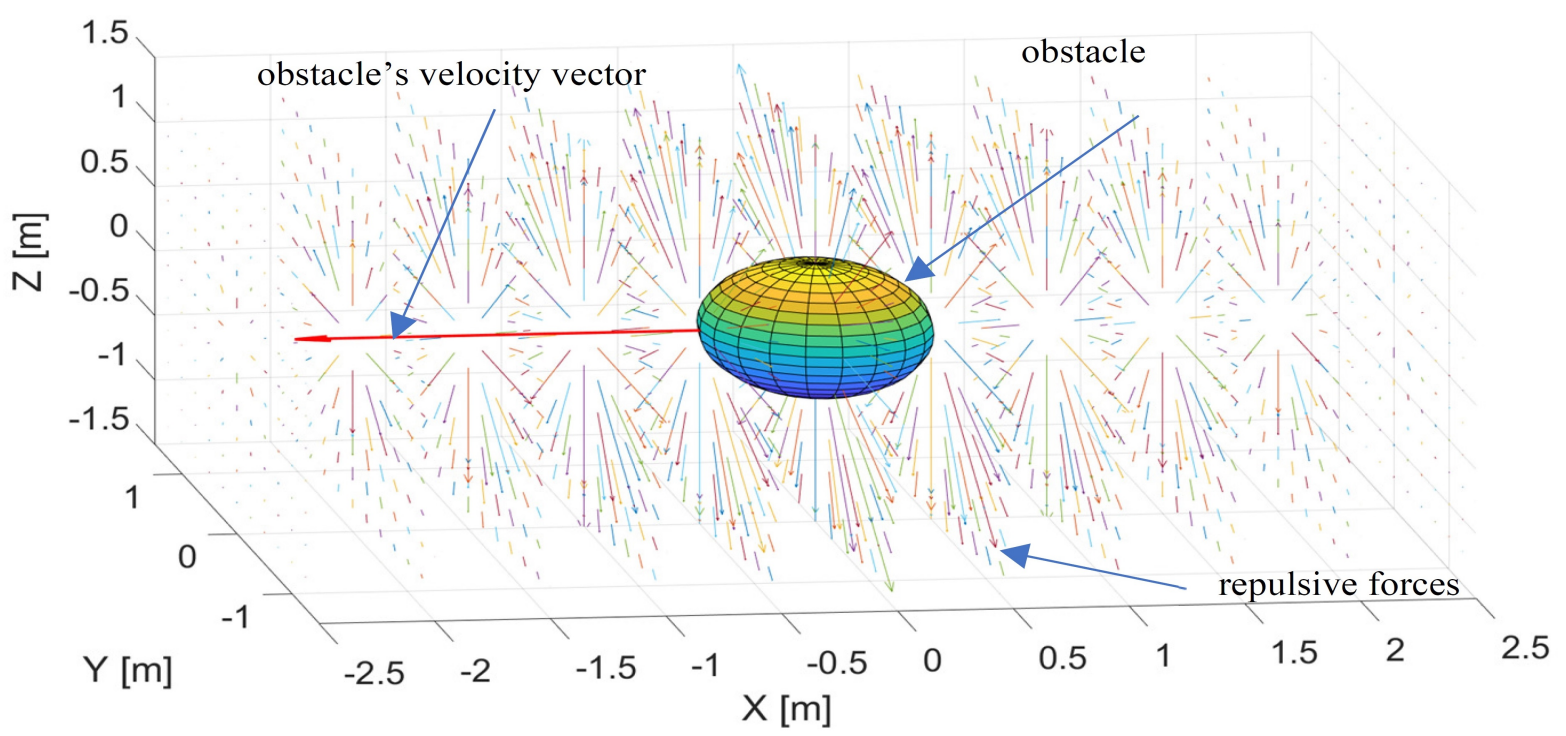
Distribution of repulsive forces defined by the multidimensional repulsive potential field $U\left(\rho_{L},\;\rho_{O},\;V_{R}\right)$.

Nonholonomic constraints resulting from motion dynamics significantly limit the maneuverability of fixed-wing UAVs [22]. Their minimal turn radius, which depends on the airspeed and bank angle, plays a crucial role in obstacle avoidance in unknown environments, especially ones that are dynamic [23,24]. Algorithms that provide nonholonomic vehicles with the ability to avoid collisions by maintaining a safe distance should, such as the repulsive fractional field described in [25], be sensitive to the relative speed and travel direction of a moving obstacle and modify the UAV path so that it passes it at a sufficient distance [26,27]. This distance must guarantee that it has enough space to make the turn even at its minimum turning radius. The main limitation of the potential field approach to obstacle avoidance is the existence of local minima where repulsive forces are canceled out by attraction forces resulting in several equilibria and oscillations [28]. This article, therefore, unlike commonly used approaches to potential fields or bipolar navigational functions [29] for obstacle avoidance path planning, proposes a repulsive potential field that is primarily dedicated to nonholonomic vehicles such as fixed-wing UAVs and produces forces repulsing them not directly from the obstacle, but perpendicularly from a line defined by its velocity vector. The strength of the repulsive force depends on relative distances from both the obstacle and the line as well as the obstacle’s speed and increases progressively when those distances decrease. The areas within which repulsive forces act on the UAV are located at the obstacle’s front and behind it. Such a repulsive potential field can, therefore, be considered multidimensional since it is not only a function of a relative distance between the UAV and the obstacle. The idea of the multidimensional repulsive potential field is explained in Figure 1.

Repulsive forces act within a plane that is perpendicular to the line of the obstacle’s velocity vector and are defined by modifying the repulsive field (determined by (1)), proposed by Khatib [7] and limited to a 2D plane. Their distribution and strength are symmetrical in relation to the line because the purpose of repulsive forces is to redirect the UAV from the area around the line defined by the obstacle’s current movement direction. The strength of repulsive forces decreases gradually when the distance to the obstacle increases along the line, symmetrically, at its front and behind it. When the UAV reaches the border of a repulsion zone where the repulsive force is the smallest, it decelerates; thus, retaining the ability to turn away within a smaller turning distance. This property of the multidimensional repulsive potential function is beneficial for nonholonomic unmanned aerial vehicles.

(1) $$U\left(\rho \right)=\left\{\begin{array}{ll} \frac{1}{2}\cdot \eta \cdot {\left(\frac{1}{\rho }-\frac{1}{\rho_{min}}\right)}^{2} & \rho \leq \rho_{min}\\ 0 & \rho >\rho_{min} \end{array},\right.$$

where ρ—the distance between the UAV and an obstacle; $\rho_{min}$—the minimum safe distance from an obstacle; η—the gain coefficient.

To determine a definition of the proposed multidimensional repulsive potential function, it was necessary to establish geometrical relations between the nonholonomic UAV and an obstacle. If the obstacle’s position in 3D space is represented as $P_{Ob}=\left[x_{Ob},\;y_{Ob},\;z_{Ob}\right]$ and its velocity vector is defined as $V_{Ob}=\left[v_{Ob}^{x},\;v_{Ob}^{y},\;v_{Ob}^{z}\right]$, then the line of its velocity vector can be defined using the following equation:

(2) $$\frac{x-x_{Ob}}{v_{Ob}^{x}}=\frac{y-y_{Ob}}{v_{Ob}^{y}}=\frac{z-z_{Ob}}{v_{Ob}^{z}}.$$

Let the UAV’s position be defined as $P_{UAV}=\left[x_{UAV},\;y_{UAV},\;z_{UAV}\right]$; then, the plane which is perpendicular to the line (2) and intersects $P_{UAV}$ is defined in the following manner:

(3) $$v_{Ob}^{x}\cdot \left(x-x_{UAV}\right)+v_{Ob}^{y}\cdot \left(y-y_{UAV}\right)+v_{Ob}^{z}\cdot \left(z-z_{UAV}\right)=0.$$

The plane from the above Equation (3) includes the distribution of repulsive forces situated around the line defined by the obstacle’s velocity vector. Equations (2) and (3) create a system of equations whose solutions establish a point at which plane (3) intersects the line (2). This point, named $P_{C}=\left[x_{C},y_{C},z_{C}\right]$, was used to determine the UAV’s distance from the obstacle and regulate the strength of repulsive forces along the line (2). Coordinates of $P_{C}$, determined on the basis of (2) and (3), were given as:

(4) $$\begin{array}{ccc} x_{C} & =\frac{{\left(v_{Ob}^{x}\right)}^{2}\cdot x_{UAV}+x_{Ob}\cdot \left({\left(v_{Ob}^{y}\right)}^{2}+{\left(v_{Ob}^{z}\right)}^{2}\right)}{\left({\left(v_{Ob}^{x}\right)}^{2}+{\left(v_{Ob}^{y}\right)}^{2}+{\left(v_{Ob}^{z}\right)}^{2}\right)}+\frac{v_{Ob}^{x}\cdot v_{Ob}^{y}\cdot \left(y_{UAV}-y_{Ob}\right)+v_{Ob}^{x}\cdot v_{Ob}^{z}\cdot \left(z_{UAV}-z_{Ob}\right)}{\left({\left(v_{Ob}^{x}\right)}^{2}+{\left(v_{Ob}^{y}\right)}^{2}+{\left(v_{Ob}^{z}\right)}^{2}\right)} & \; \end{array},$$

(5) $$\begin{array}{ccc} y_{C} & =\frac{{\left(v_{Ob}^{y}\right)}^{2}\cdot y_{UAV}+y_{Ob}\cdot \left({\left(v_{Ob}^{x}\right)}^{2}+{\left(v_{Ob}^{z}\right)}^{2}\right)}{\left({\left(v_{Ob}^{x}\right)}^{2}+{\left(v_{Ob}^{y}\right)}^{2}+{\left(v_{Ob}^{z}\right)}^{2}\right)}+\frac{v_{Ob}^{x}\cdot v_{Ob}^{y}\cdot \left(x_{UAV}-x_{Ob}\right)+v_{Ob}^{y}\cdot v_{Ob}^{z}\cdot \left(z_{UAV}-z_{Ob}\right)}{\left({\left(v_{Ob}^{x}\right)}^{2}+{\left(v_{Ob}^{y}\right)}^{2}+{\left(v_{Ob}^{z}\right)}^{2}\right)} & \; \end{array},$$

(6) $$\begin{array}{ccc} z_{C} & =\frac{{\left(v_{Ob}^{z}\right)}^{2}\cdot z_{UAV}+z_{Ob}\cdot \left({\left(v_{Ob}^{x}\right)}^{2}+{\left(v_{Ob}^{y}\right)}^{2}\right)}{\left({\left(v_{Ob}^{x}\right)}^{2}+{\left(v_{Ob}^{y}\right)}^{2}+{\left(v_{Ob}^{z}\right)}^{2}\right)}+\frac{v_{Ob}^{x}\cdot v_{Ob}^{z}\cdot \left(x_{UAV}-x_{Ob}\right)+v_{Ob}^{y}\cdot v_{Ob}^{z}\cdot \left(y_{UAV}-y_{Ob}\right)}{\left({\left(v_{Ob}^{x}\right)}^{2}+{\left(v_{Ob}^{y}\right)}^{2}+{\left(v_{Ob}^{z}\right)}^{2}\right)} & \; \end{array}.$$

Having defined points $P_{C}$, $P_{Ob},$ and $P_{UAV}$, it was possible to calculate distances between the UAV and the line (2) in plane (3), as well as those between the UAV and the obstacle along this line. We expressed them using the following symbols: $\rho_{L}$—the distance to the line (2) in plane (3); $\rho_{O}$—the distance to the obstacle along line (2). Both distances were used in the repulsive potential field definition and they were represented by the following formula:

(7) $$\rho_{L}=\sqrt{{\left(x_{UAV}-x_{C}\right)}^{2}+{\left(y_{UAV}-y_{C}\right)}^{2}+{\left(z_{UAV}-z_{C}\right)}^{2}},$$

(8) $$\rho_{O}=\sqrt{{\left(x_{C}-x_{Ob}\right)}^{2}+{\left(y_{C}-y_{Ob}\right)}^{2}+{\left(z_{C}-z_{Ob}\right)}^{2}}.$$

The original function of the repulsive potential field proposed in [7] had a constant slope around the point of its maximum and the lengths of its gradients were, therefore, dependent only on relative distance ρ. In our approach, we used the potential field (1) to create a field of repulsive forces in-plane (3) around point $P_{C}$. This field should not only be a function of $\rho_{L}$, but also a function of distance $\rho_{O}$ and relative speed $V_{R}$ along line (2). Only then was the proposed repulsive potential field multidimensional and became applicable in environments with dynamic obstacles. We defined the repulsive potential function as follows:

(9) $$U\left(\rho_{L},\;a\right)=\left\{\begin{array}{ll} \frac{1}{2}\cdot \eta \cdot a\cdot {\left(\frac{1}{\left(\rho_{L}+1\right)}-\frac{1}{\left(\rho_{Lmin}+1\right)}\right)}^{2} & \rho_{L}\leq \rho_{Lmin}\\ 0 & \rho_{L}>\rho_{Lmin} \end{array},\right.$$

where *a*—a function of $\rho_{O}$ and $V_{R}$, which means that the maximum of function (9), i.e., $U\left(0,\;a\right)=\frac{a\cdot \rho_{Lmin}}{2\cdot \left(\rho_{Lmin}+1\right)}$, is also a function of $\rho_{O}$ and $V_{R}$; $\rho_{Lmin}$—the minimum distance to line (2).

**Figure 2:**
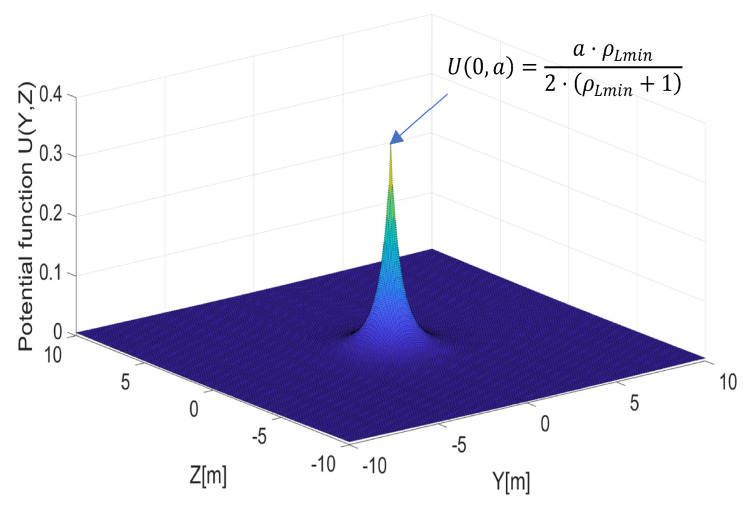
The repulsive potential function $U\left(\rho_{L},\;a\right)$ from (9).

In this way, the slope of potential function (9) was regulated by $\rho_{O}$ and $V_{R}$ and was not constant as happened with (1). Since the turn radius (angular rate of heading angle) of a nonholonomic fixed-wing UAV is a reversely proportional function of speed (22), the repulsive potential should also be a reverse function of $V_{R}$. The reasoning is that even if relative speed $V_{R}\;$ is high, the UAV should decelerate to achieve a lower turn radius, allowing it to avoid the area around the obstacle’s path. The repulsive potential function, given as (9), creates a distribution of repulsive forces in plane (3) as shown in Figure 2. Figure 2 presents the shape of the function (9).

Parameter *a* is a simple proportional function $V_{R}$ and an inverse proportional function of $\rho_{O}$:

(10) $$a\left(\rho_{O},\;V_{R}\right)=\left\{\begin{array}{ll} \frac{1}{V_{R}+1}\cdot \left(\rho_{Omin}-\rho_{O}\right) & \rho_{O}<\rho_{Omin}\;\cap \;V_{R}\neq 0\\ 0 & \rho_{O}\geq \rho_{Omin}\;\cup \;V_{R}=0 \end{array},\right.\;$$

where $\rho_{Omin}$—minimum safe distance to obstacle along line (2) at the front and behind.

Substituting (10) into (9) results in equation:

(11) $$\begin{array}{cccc} \; & U\left(\rho_{L},\;\rho_{O},\;V_{R}\right)=\; & \left\{\begin{array}{ll} \frac{1}{2}\eta \frac{1}{V_{R}+1}\left(\rho_{Omin}-\rho_{O}\right)\;{\left(\frac{1}{\left(\rho_{L}+1\right)}-\frac{1}{\left(\rho_{Lmin}+1\right)}\right)}^{2}, & \rho_{L}\leq \rho_{Lmin}\;{\cap \;\rho }_{O}<\rho_{Omin}\;\cap \;V_{R}\neq 0\\ 0,\rho_{L}>\rho_{Lmin}\cup \rho_{O}\geq \rho_{Omin}\;\cup \;V_{R}=0 \end{array}\right. & \; \end{array}.$$

The multidimensional repulsive potential function is dependent on the relative speed $V_{R}\;$ between the UAV and the obstacle, given in the direction of velocity vector $V_{Ob}$. Therefore, to calculate $V_{R}$, it was necessary to project the UAV’s velocity vector $V_{UAV}$ perpendicularly onto line (2). If we allowed $V_{UAV}^{p}$ to be a vector that was a perpendicular projection on line (2), then the perpendicular projection of $V_{UAV}$ on $V_{Ob}$ was defined as follows:

(12) $$V_{UAV}^{p}=\frac{V_{UAV}\circ V_{Ob}}{{\left(\left|V_{Ob}\right|\right)}^{2}}\cdot V_{Ob}.$$

Because the fraction before $V_{Ob}$ is a scalar, (12) for vectors’ lengths could be written in another form.

(13) $$\left|V_{UAV}^{p}\right|=\frac{V_{UAV}\circ V_{Ob}}{{\left(\left|V_{Ob}\right|\right)}^{2}}\left|V_{Ob}\right|=\frac{V_{UAV}\circ V_{Ob}}{\left|V_{Ob}\right|}.$$

Finally, on the basis of (13), it was possible to determine $V_{R}$ as the difference between $\left|V_{UAV}^{p}\right|$ and $\left|V_{Ob}\right|$:

(14) $$\begin{array}{ccc} V_{R}\left(V_{UAV},V_{Ob}\right) & =\left|V_{UAV}^{p}\right|-\left|V_{Ob}\right|=\frac{V_{UAV}\circ V_{Ob}}{\left|V_{Ob}\right|}-\left|V_{Ob}\right| & \\ & =\frac{{v_{UAV}^{x}\cdot v}_{Ob}^{x}+{v_{UAV}^{y}\cdot v}_{Ob}^{y}+{v_{UAV}^{z}\cdot v}_{Ob}^{z}-{\left(v_{Ob}^{x}\right)}^{2}-{\left(v_{Ob}^{y}\right)}^{2}-{\left(v_{Ob}^{z}\right)}^{2}}{\sqrt{{\left(v_{Ob}^{x}\right)}^{2}+{\left(v_{Ob}^{y}\right)}^{2}+{\left(v_{Ob}^{z}\right)}^{2}}} \end{array}.$$

**Figure 3:**
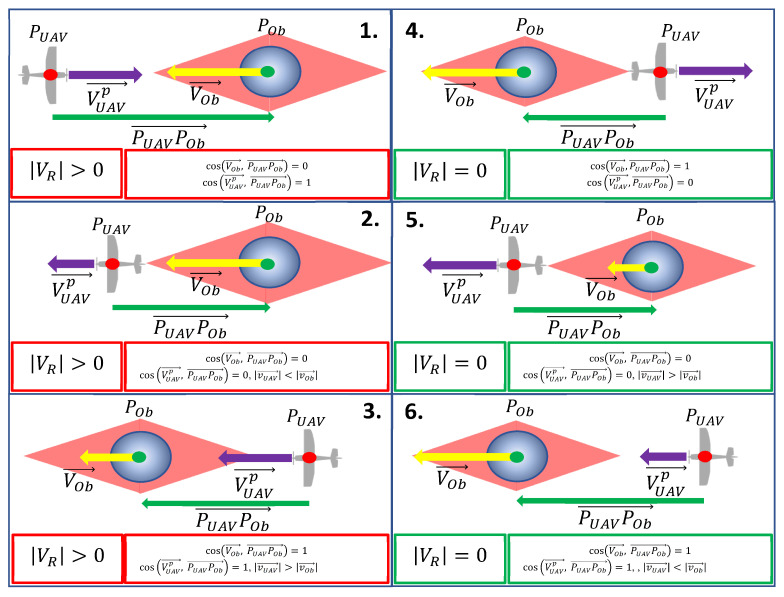
Scenarios of mutual orientation of the UAV, the obstacle, and their velocity vectors. $P_{Ob}$—the obstacle’s position (the green point);${\;P}_{UAV}$—the UAV’s position (the red point); $V_{Ob}$—the obstacle’s velocity vector (the yellow arrow); $V_{UAV}^{p}$—the UAV’s velocity vector projection on the line of $V_{Ob}$ (the violet arrow). Scenarios **1**–**3** are dangerous for the UAV when $V_{R}$ value calculated using (14) is different than 0. Scenarios **4**–**6** are safe for the UAV when $V_{R}$ value is equal to 0; thus, canceling out the repulsive force. The red area around the obstacle represents an area where the repulsive force is valid.

Because $V_{R}$ is a scalar and does not include information about spatial orientation $V_{UAV}$ in reference to $V_{Ob}$, it was necessary to evaluate whether given scenarios were dangerous or safe in respect to the UAV. Safe scenarios were those where it could be assumed that $V_{R}=0$ and where the repulsive force was canceled out. All possible scenarios of mutual orientations for $V_{Ob}$ and $V_{UAV}^{p}$ are described in Figure 3.

Constraints presented in Figure 3 were applied to (14) to regulate the strength of the repulsive force only in cases involving a risk of collision. In safe situations, the repulsive force was not necessary, i.e., if *V_R_* was zero, then $U\left(\rho_{L},\;\rho_{O},\;V_{R}\right)=0$. This concerns scenarios 4–6 presented in Figure 3, where, in the first example, the velocity vectors of the obstacle and the UAV were opposite to one another; in the second, the UAV was in front of the obstacle with both velocity vectors having the same orientation, but the UAV’s velocity vector was greater than the obstacle’s; while, in the last instance, the UAV was behind the obstacle, the orientation of both velocity vectors was also consistent, but the UAV’s velocity vector was shorter.

**Figure 4:**
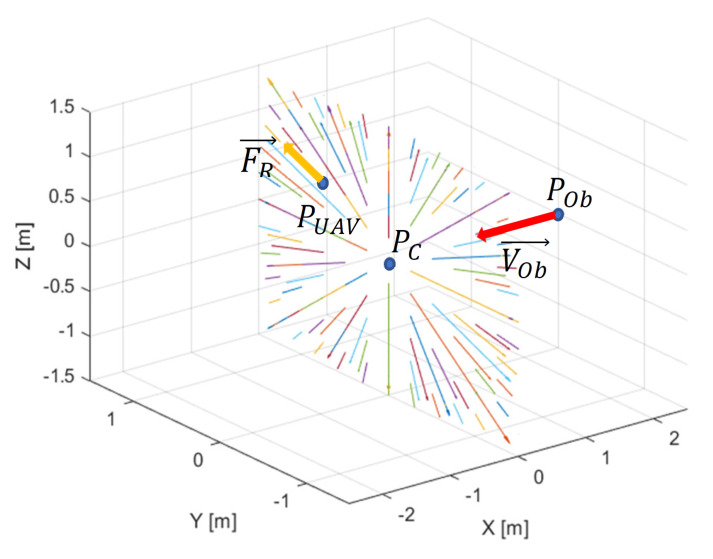
Repulsive forces around point $P_{C}$ in the plane perpendicular to the line of the obstacle’s velocity vector. $F_{R}$ repulses the UAV from $P_{C}$ lying on the line.

Repulsive forces presented in Figure 4 are reverse gradients of the multidimensional repulsive potential function from (11), represented by the following equation:

(15) $$\begin{array}{cccc} F_{R} & =\nabla U\left(P_{UAV},P_{Ob},P_{C}\right)=\eta \cdot \left(V_{R}+1\right)\cdot \left(\rho_{Omin}-\rho_{O}\left(P_{Ob},P_{C}\right)\right)\cdot \left(\frac{1}{\left(\rho_{L}\left(P_{UAV},P_{C}\right)+1\right)}-\frac{1}{\left(\rho_{Lmin}+1\right)}\right)\cdot & \; & \\ \; & \frac{1}{{\left(\rho_{L}\left(P_{UAV},\;P_{C}\right)+1\right)}^{2}}\cdot \left(P_{UAV}{-P}_{C}\right) \end{array},$$

where $P_{UAV}$—the UAV’s position; $P_{Ob}$—the obstacle’s positions; $P_{C}$—the position of the point of intersection of plane (3) and line (2); $\rho_{Lmin}$—the minimum distance between the UAV and the point defined as $P_{C}$; $\rho_{Omin}$—the minimum distance between the obstacle and the point defined as $P_{C}$.

Repulsive force (15) could be used to calculate the setpoints of control loops for the heading angle, pitch angle, and speed. The following equation could be used to find desired values of the heading, pitch angles, and speed [30]:

(16) $$\psi_{D}=atan2\left(F_{R}\left(y\right),F_{R}\left(x\right)\right),$$

(17) $$\theta_{D}=atan2\left(F_{R}\left(z\right),\sqrt{{F_{R}\left(x\right)}^{2}+{F_{R}\left(y\right)}^{2}}\right),$$

(18) $$V_{D}=\sqrt{{F_{R}\left(x\right)}^{2}+{F_{R}\left(y\right)}^{2}+{F_{R}\left(z\right)}^{2}},$$

where $\psi_{D}$—the desired heading angle; $\theta_{D}$—the desired pitch angle; $V_{D}$—the desired speed.

The next section describes numerical simulations that were carried out to verify obstacle avoidance based on repulsive forces (15).

## 3. Numerical Simulations of Typical Collision Scenarios

To assess the possibilities of the proposed repulsive potential function and the effectiveness of its repulsive forces in achieving safe obstacle avoidance by nonholonomic UAVs, numerical simulations were prepared on the basis of a 3D nonholonomic model of the UAV. In this model, coordinates of the unmanned aerial vehicle were plotted onto a Cartesian frame and the flight was controlled by setpoints of the heading angle $\psi_{D}$, pitch angle $\theta_{D}$, and speed $V_{D}$. The model’s system of equations was as follows [28,31,32]:

(19) $$\dot{x}=V\cdot cos\psi \cdot cos\theta ,$$

(20) $$\dot{y}=V\cdot sin\psi \cdot cos\theta ,$$

(21) $$\dot{z}=V\cdot sin\theta ,$$

(22) $$\dot{\psi }=\frac{g}{V}\cdot tan\phi ,$$

(23) $$\dot{V}=\alpha_{V}\cdot \left(V_{D}-V\right),$$

(24) $$\dot{\theta }=\alpha_{\theta }\cdot \left(\theta_{D}-\theta \right),$$

(25) $$\phi =\alpha_{\phi }\cdot \left(\psi_{D}-\psi \right),$$

where $\psi_{D}$—the desired heading angle from (16); ψ—the heading angle; $\theta_{D}$—the desired pitch angle from (17); θ—the pitch angle; $V_{D}$—the desired speed from (18); *V*—the speed; ϕ—the bank angle; *x*,*y*,*z*—the coordinates of the UAV within the Cartesian frame; $\alpha_{V}$, $\alpha_{\theta }$, $\alpha_{\phi }$—coefficients having the weight of time constants of inertia.

**Figure 5:**
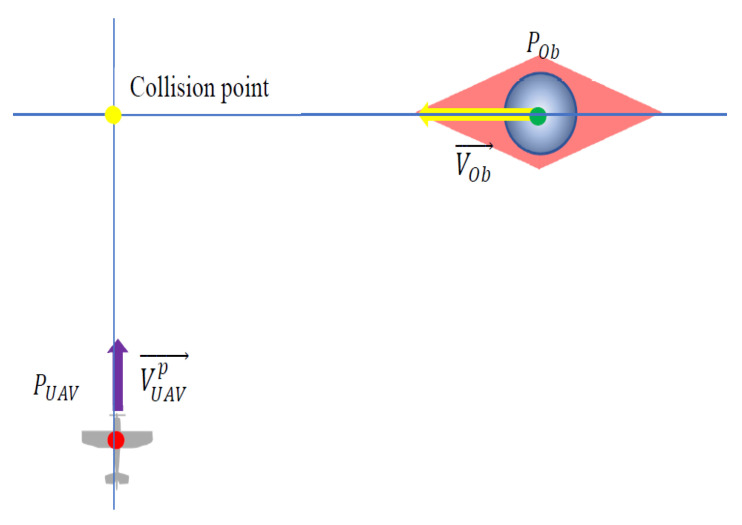
A collision scenario where the UAV and the obstacle meet each other at a point where their paths intersect.

The model described by (19)–(25) is a driftless system that cannot be stabilized at setpoints using smooth time-invariant feedback [33]. This complicated the navigation problem with respect to nonholonomic vehicles, especially in situations where dynamic obstacles impose state vector constraints on them. However, the proposed repulsive potential should simplify the problem. The repulsive force became a time-variant setpoint, a function of relative speed and position of the UAV locating it within zones at the front of and behind the obstacle. Outside these zones, the setpoint was controlled by waypoint navigation. The purpose of the repulsive potential function was, of course, to ensure flight safety among obstacles and not stabilization; thus, entering into a zone of repulsive forces could cause a violent change of the setpoint. To verify the effectiveness of the repulsive potential function in keeping the UAV away from moving obstacles and to assess its impact on flight stability, three different scenarios containing the threat of collision with a single obstacle were simulated. The first situation was critical since, the UAV and the obstacle were heading directly towards one another (Figure 3(1)). In the second scenario, the UAV was closing in on the obstacle from behind (Figure 3(3)), while, in the last one, the paths of the UAV and the obstacle intersected perpendicularly (Figure 5). In the scenario depicted in Figure 5, the speeds of the UAV and the obstacle, as well as their directions of travel from their initial positions, were established so that the UAV and the obstacle would meet at a point where their paths intersected. This situation may be critical because the spatial orientation of the repulsive force was exactly opposite to the UAV’s velocity vector, while its turn radius was limited by its nonholonomic constraints.

In each of the three scenarios, the values of minimal distances $\rho_{Omin}$, $\rho_{Lmin}$, as well as those of the obstacle’s speed, were varied to observe their impact on collision avoidance defined by the length of the shortest distance between the UAV and the obstacle.

## 4. Results

**Figure 6:**
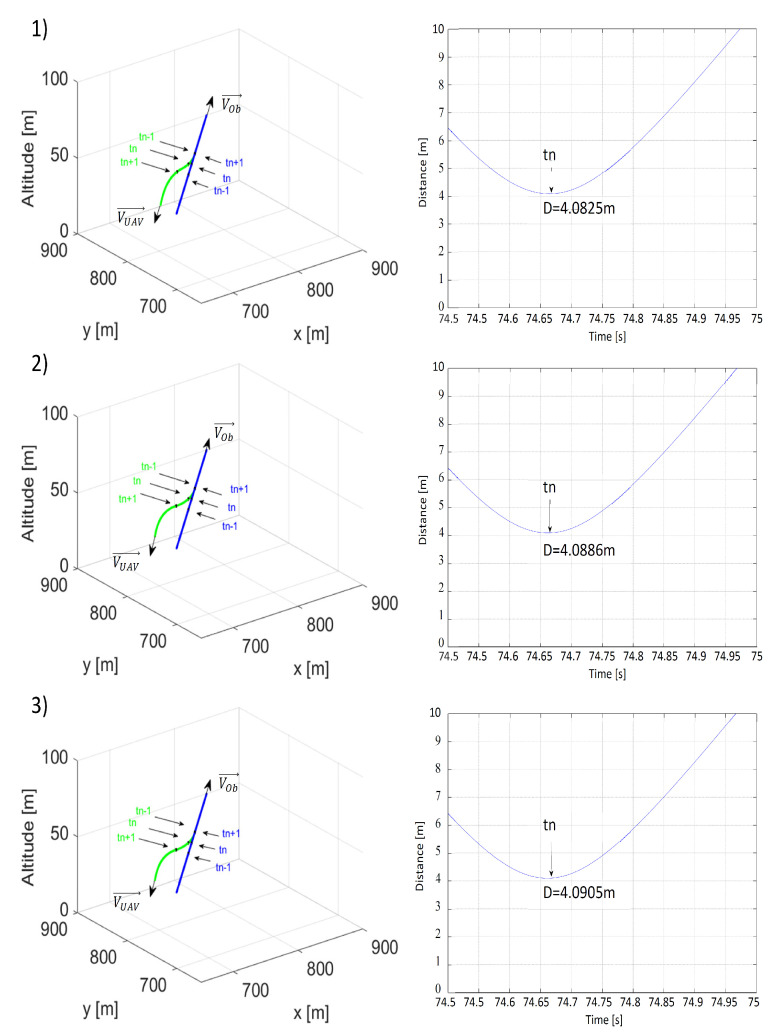
Time plots of paths (the UAV—green; the obstacle—blue) and distances between the UAV and the obstacle for (**1**) $\rho_{Lmin}$ = 10 m, $\rho_{Omin}$ = 50 m, $\left|V_{Ob}\right|=\left|V_{UAV}\right|=15$ m/s; (**2**) $\rho_{Lmin}$ = 20 m, $\rho_{Omin}$ = 50 m, $\left|V_{Ob}\right|=\left|V_{UAV}\right|=15$ m/s; (**3**) $\rho_{Lmin}$ = 30 m, $\rho_{Omin}$ = 50 m, $\left|V_{Ob}\right|=\left|V_{UAV}\right|=15$ m/s. $t_{n}$—the moment when the distance was the shortest; $t_{n-1}$—the moment 1 s before $t_{n}$; $t_{n+1}$—the moment 1 s after $t_{n}$.

**Figure 7:**
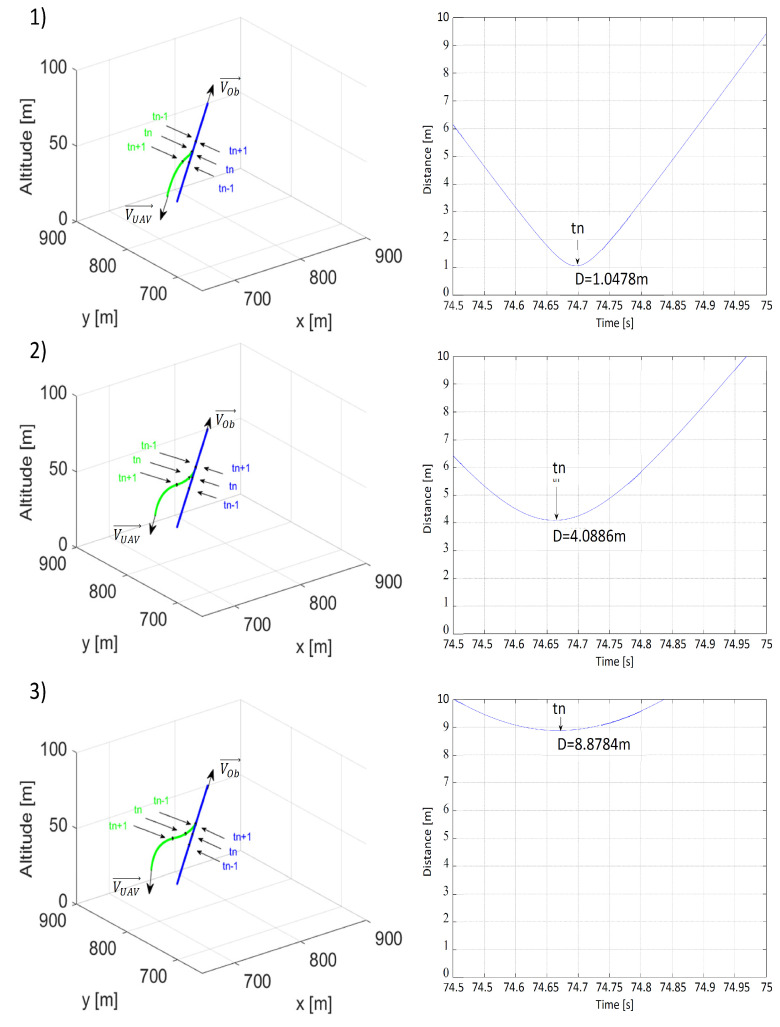
Time plots of paths (the UAV—green; the obstacle—blue) and distances between the UAV and the obstacle for (**1**) $\rho_{Lmin}$ = 20 m, $\rho_{Omin}$ = 25 m, $\left|V_{Ob}\right|=\left|V_{UAV}\right|=15$ m/s; (**2**) $\rho_{Lmin}$ = 20 m, $\rho_{Omin}$ = 50 m, $\left|V_{Ob}\right|=\left|V_{UAV}\right|=15$ m/s; (**3**) $\rho_{Lmin}$ = 20 m, $\rho_{Omin}$ = 75 m, $\left|V_{Ob}\right|=\left|V_{UAV}\right|=15$ m/s. $t_{n}$—the moment when the distance was the shortest; $t_{n-1}$—the moment 1 s before $t_{n}$; $t_{n+1}$—the moment 1 s after $t_{n}$.

**Figure 8:**
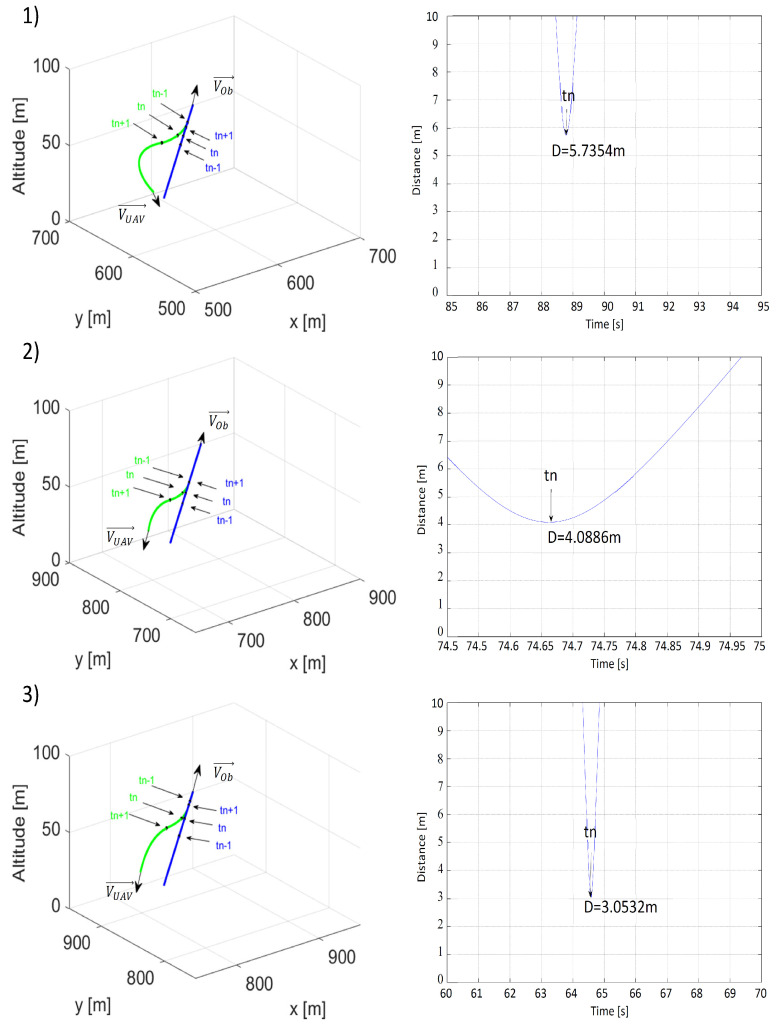
Time plots of paths (the UAV—green; the obstacle—blue) and distances between the UAV and the obstacle for (**1**) $\rho_{Lmin}$ = 20 m, $\rho_{Omin}$ = 50 m, $\left|V_{Ob}\right|=1$0 m/s; (**2**) $\rho_{Lmin}$ = 20 m, $\rho_{Omin}$ = 50 m, $\left|V_{Ob}\right|=15$ m/s; (**3**) $\rho_{Lmin}$ = 20 m, $\rho_{Omin}$ = 50 m, $\left|V_{Ob}\right|=20$ m/s. $t_{n}$—the moment when the distance was the shortest; $t_{n-1}$—the moment 1 s before $t_{n}$; $t_{n+1}$—the moment 1 s after $t_{n}$.

In the first scenario, where the UAV and the obstacle were moving directly towards one another, their initial positions were, respectively, $P_{UAV0}=\left[1500,\;1550,\;50\right]$ and $P_{Ob0}=\left[0,\;50,\;50\right]$. The UAV’s speed was constant and was equal to $\left|V_{UAV}\right|$ = 15 m/s. Initial heading angles for the UAV and the obstacle were as follows: $\psi_{Ob}=45^{\circ }$ and $\psi_{UAV}=225^{\circ }$. For the obstacle, its heading angle was constant, while the UAV’s heading angle was controlled by repulsive forces within the area surrounding the obstacle. Coefficients of the 3D nonholonomic model of the UAV were $\alpha_{V}$ = 0.25, $\alpha_{\theta }$ = 0.5, and $\alpha_{\phi }$ = 0.5. The value of the maximum bank angle was limited to ±17${}^{\circ }$, a typical value for fixed-wing UAVs. The following values of multidimensional repulsive function parameters were used in the simulations: $\eta =10,\;\rho_{Omin}$ = 25, 50, 75 m, $\rho_{Lmin}$ = 10, 20 or 30 m, and $\left|V_{Ob}\right|$ = 10, 15 or 20 m/s. Simulated flight paths and time plots of distances between the UAV and the obstacle are presented in Figure 6, Figure 7 and Figure 8. Each figure concerns the analysis of the influence of a different coefficient of the repulsive potential function on the safety of the resulting obstacle avoidance maneuver.

According to Figure 6, when it came to a frontal collision, it could be concluded that the value of $\rho_{Lmin}$ did not significantly impact the smallest value of distance between the UAV and the obstacle. When distance $\rho_{Lmin}$ was increased from 10 m to 30 m, the result was a less than 1 cm increase in that distance. At the same time, we could observe that the distance between flight paths increased after collision avoidance more for $\rho_{Lmin}$ = 30 than for $\rho_{Lmin}$ = 10. A comparison of flight paths from Figure 6(1,3) showed this clearly and proved that the repulsive force increased with $\rho_{Lmin}$—the minimal distance from the line of the obstacle’s velocity vector.

Figure 7 compares the paths and minimal distances between the UAV and the obstacle at different values of minimal distance $\rho_{Omin}\;$ along the line of the obstacle’s velocity vector. In accordance with the author’s predictions, just as in the simulation involving a threat of a frontal collision, the minimal collision avoidance distance was strongly dependent on the value of $\rho_{Omin}$. Changing $\rho_{Omin}$ from 25 m to 75 m results in an increase in the minimal distance from 1.0478 to 8.8784 m.

Figure 8 presents plots of paths and time plots of distances between the UAV and the obstacle at different values of the obstacle’s speed. The minimum collision avoidance distance decreased as the obstacle’s speed and the relative speed $V_{R}$ increased. The inertia of the UAV’s response to the repulsive force canceled out the effects of the rapid desired heading angle change and deceleration induced by the repulsive potential field.

The repulsive force was smallest when the UAV crossed the boundaries of repulsion zones located in front of and behind the obstacle, and distance $\rho_{L}$ fell below $\rho_{Lmin}$ or distance $\rho_{O}$ reduced to below $\rho_{Omin}$. Since it was perpendicular to the obstacle’s as well as the UAV’s velocity vectors, it caused the UAV to decelerate and perform a 90${}^{\circ }$ turn. However, due to its inertia, the UAV was unable to reduce its turning radius through deceleration and the utilization of its maximum bank angle to conduct the turn as quickly as it was necessary to avoid a collision, illustrating the fact that a UAV’s nonholonomic constraints are of crucial significance when it comes to situations carrying the risk of a frontal collision.

In conclusion, when it comes to cases where frontal collision is imminent, the most important parameter of the multidimensional repulsive function is the minimal distance of the UAV to the obstacle $\rho_{Omin}$. Its value should provide the UAV with enough space between it and the obstacle to allow it to make the turn at a maximum relative speed.

**Figure 9:**
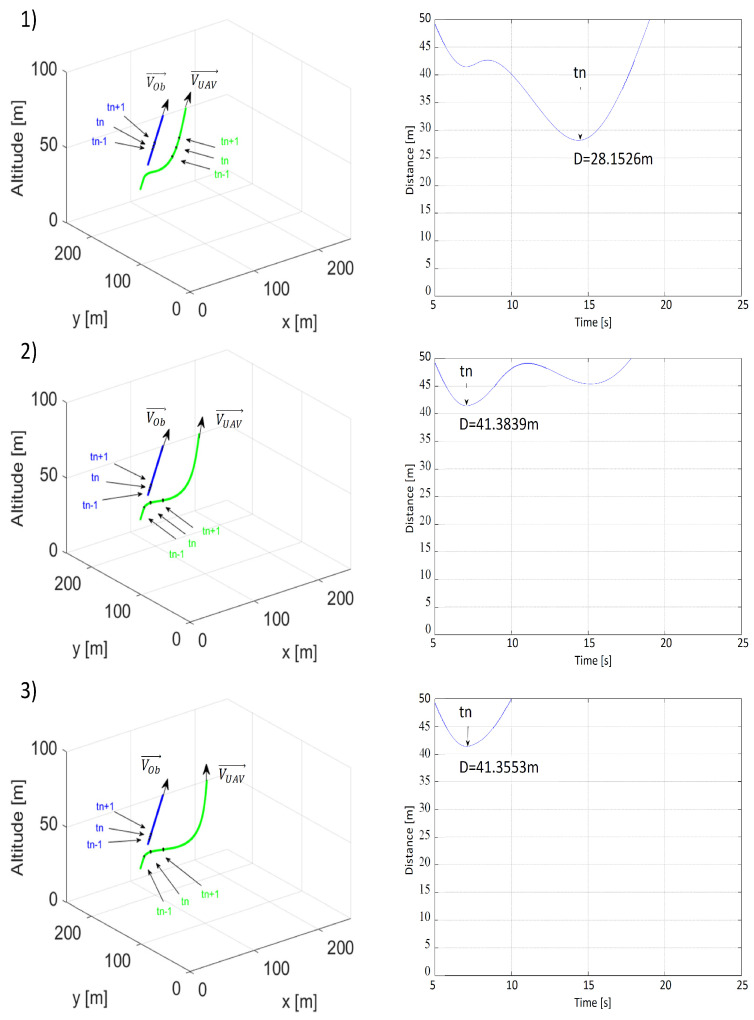
Time plots of paths (the UAV—green; the obstacle—blue) and distances between the UAV and the obstacle for (**1**) $\rho_{Lmin}$ = 10 m, $\rho_{Omin}$ = 50 m, $\left|V_{Ob}\right|=5,\left|V_{UAV}\right|=15$ m/s; (**2**) $\rho_{Lmin}$ = 20 m, $\rho_{Omin}$ = 50 m, $\left|V_{Ob}\right|=5,\left|V_{UAV}\right|=15$ m/s; (**3**) $\rho_{Lmin}$ = 30 m, $\rho_{Omin}$ = 50 m, $\left|V_{Ob}\right|=5,\left|V_{UAV}\right|=15$ m/s. $t_{n}$—the moment when the distance was the shortest; $t_{n-1}$—the moment 1 s before $t_{n}$; $t_{n+1}$—the moment 1 s after $t_{n}$.

**Figure 10:**
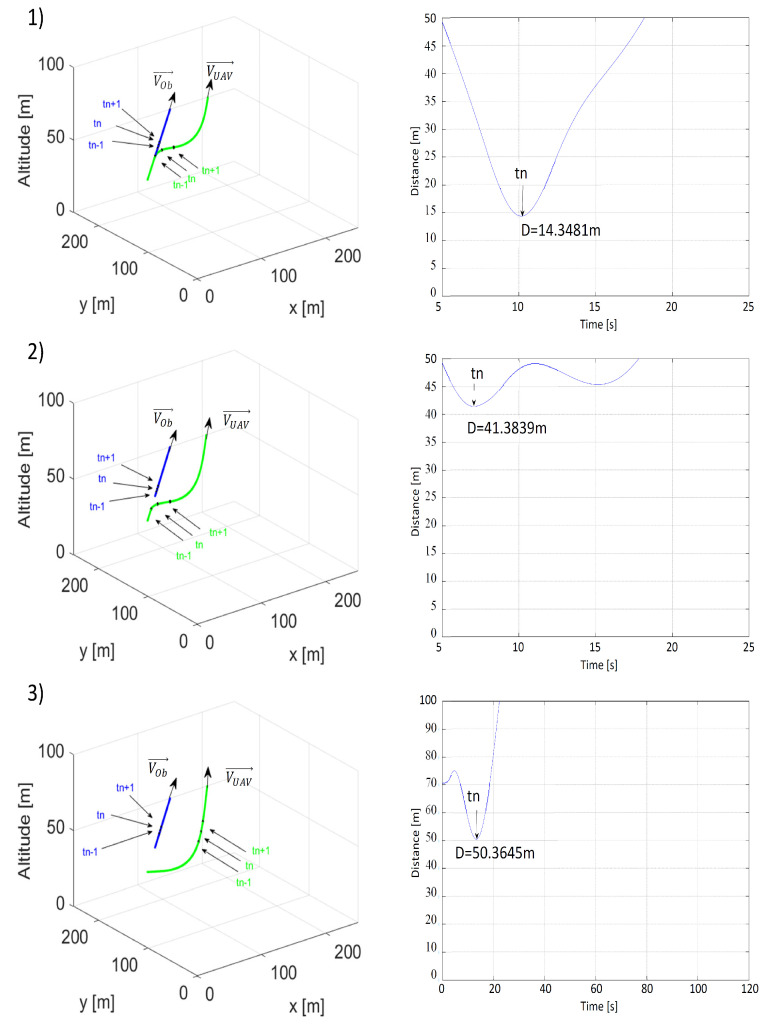
Time plots of paths (the UAV—green; the obstacle—blue) and distances between the UAV and the obstacle for (**1**) $\rho_{Lmin}$ = 20 m, $\rho_{Omin}$ = 25 m, $\left|V_{Ob}\right|=5,\left|V_{UAV}\right|=15$ m/s; (**2**) $\rho_{Lmin}$ = 20 m, $\rho_{Omin}$ = 50 m, $\left|V_{Ob}\right|=5,\left|V_{UAV}\right|=15$ m/s; (**3**) $\rho_{Lmin}$ = 20 m, $\rho_{Omin}$ = 75 m, $\left|V_{Ob}\right|=5,\left|V_{UAV}\right|=15$ m/s. $t_{n}$—the moment when the distance was the shortest; $t_{n-1}$—the moment 1 s before $t_{n}$; $t_{n+1}$—the moment 1 s after $t_{n}$.

**Figure 11:**
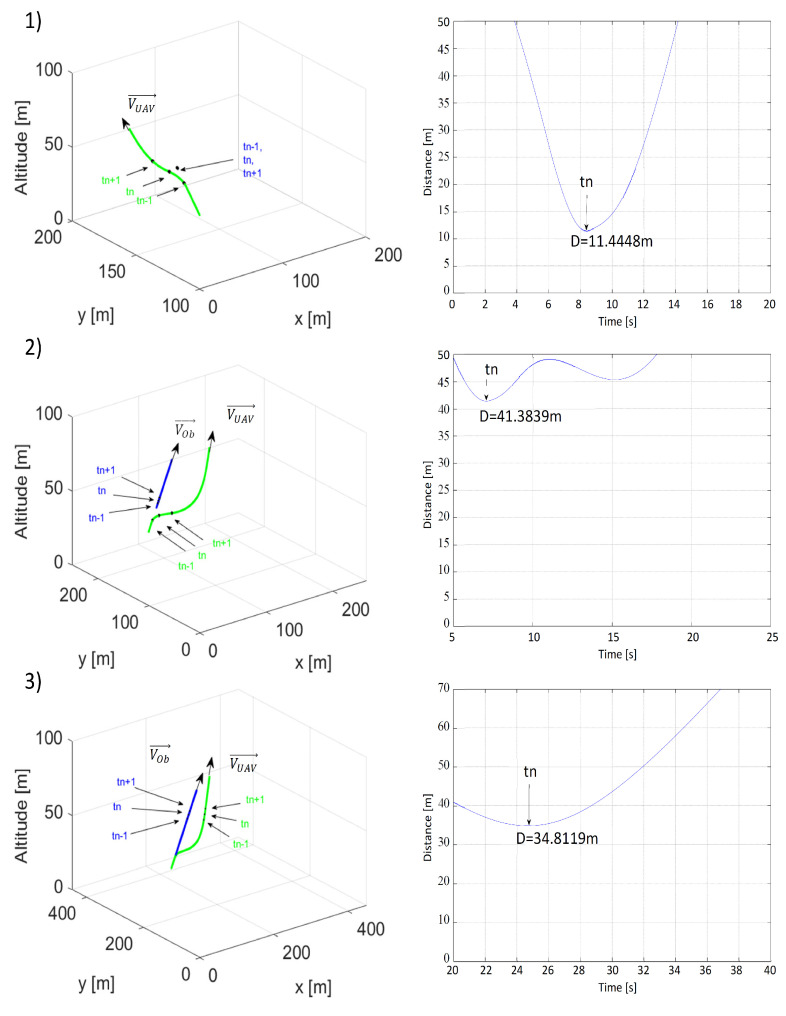
Time plots of paths (the UAV—green; the obstacle—blue) and distances between the UAV and the obstacle for (**1**) $\rho_{Lmin}$ = 20 m, $\rho_{Omin}$ = 50 m, $\left|V_{Ob}\right|=0,\left|V_{UAV}\right|=15$ m/s; (**2**) $\rho_{Lmin}$ = 20 m, $\rho_{Omin}$ = 50 m, $\left|V_{Ob}\right|=5,\left|V_{UAV}\right|=15$ m/s; (**3**) $\rho_{Lmin}$ = 20 m, $\rho_{Omin}$ = 50 m, $\left|V_{Ob}\right|=10,\left|V_{UAV}\right|=15$ m/s. $t_{n}$—the moment when the distance was the shortest; $t_{n-1}$—the moment 1 s before $t_{n}$; $t_{n+1}$—the moment 1 s after $t_{n}$.

In the second scenario, the one assuming that the UAV was flying behind the obstacle with both moving in the same direction, identical parameters’ values or their combinations were used. In this scenario, to simulate collision avoidance, changes were made only to the initial positions, speeds, and heading angles: $P_{UAV0}=\left[0,\;100,\;50\right]$, $P_{Ob0}=\left[50,\;150,\;50\right]$, $\psi_{Ob}$= $\psi_{UAV}=45^{\circ }$, $\left|V_{UAV}\right|$ = 15 m/s, and $\left|V_{Ob}\right|$ = 0, 5 or 10 m/s. Figure 9, Figure 10 and Figure 11 present simulation results.

In this scenario, distance $\rho_{Lmin}$ played a more important role in collision avoidance than in the previous scenario, because the paths of the UAV and the obstacle moved away from each other as the value of $\rho_{Lmin}$ increased. Despite this, at time *t_n_*, the distance from the obstacle to the UAV was shorter in Figure 9(3) than in Figure 9(2). Increasing $\rho_{Lmin}$ from 10 to 20 m resolved this issue and the distance in Figure 10(3) became significantly greater than in Figure 10(2). Distance $\rho_{Omin}$, as in the first scenario, prompted the UAV to make the turn earlier; thus, increasing the minimal distance along with $\rho_{Omin}$.

The most interesting results are presented in Figure 11, which analyzed collision avoidance for different obstacle speed values and concerned mainly the avoidance of a stationary obstacle. The results of this simulation indicated that the multidimensional repulsive function was also effective with these types of obstacles. When $\left|V_{Ob}\right|$ = 0 coordinates of point *P*${}_{C}$ were the same as the obstacle’s *P_O_*, and distance $\rho_{O}=0$. If it was assumed that *P_C_* = *P_Ob_*, $\rho_{O}=0$ and $\left|V_{Ob}\right|$ = 0, the repulsive potential function (11) would create a spherical repulsive force field around the obstacle. The main advantage of the multidimensional repulsive potential function was its flexibility. For $\left|V_{Ob}\right|$ = 10 m/s, during the 25th second of the simulation, the distance between the UAV and the obstacle was lower than in Figure 11(2), because the UAV overtook the obstacle when they were moving parallel to one another and the repulsive force was weak.

**Figure 12:**
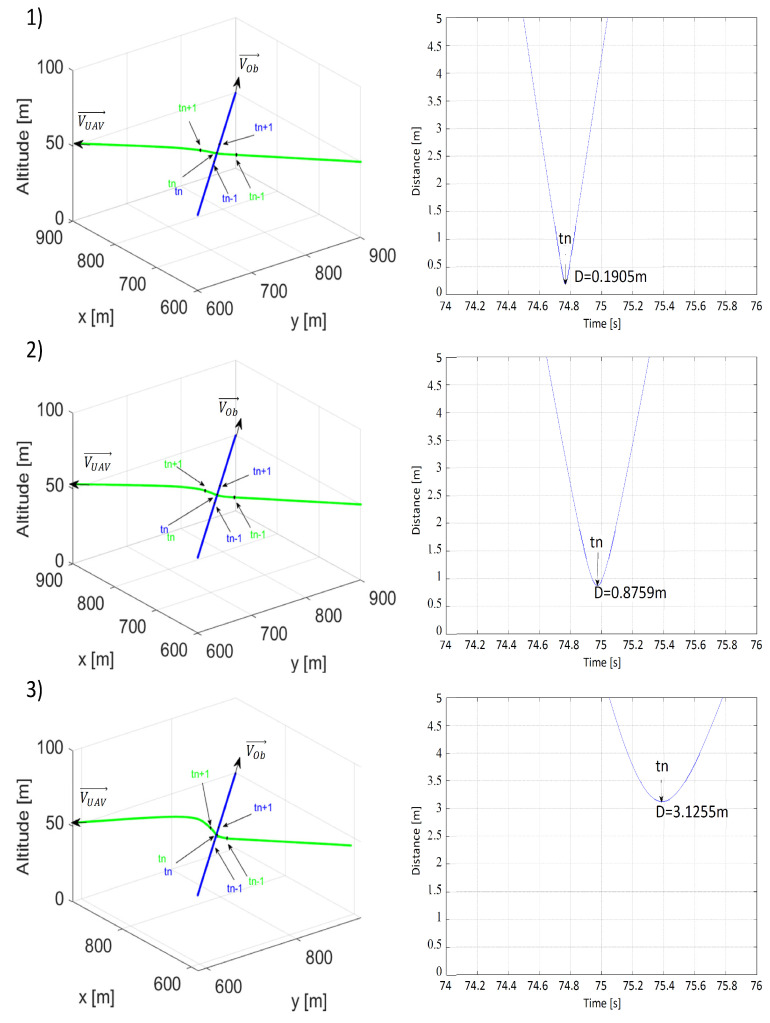
Time plots of paths (the UAV—green; the obstacle—blue) and distances between the UAV and the obstacle for (**1**) $\rho_{Lmin}$ = 10 m, $\rho_{Omin}$ = 50 m, $\left|V_{Ob}\right|=\left|V_{UAV}\right|=15$ m/s; (**2**) $\rho_{Lmin}$ = 20 m, $\rho_{Omin}$ = 50 m, $\left|V_{Ob}\right|=\left|V_{UAV}\right|=15$ m/s; (**3**) $\rho_{Lmin}$ = 30 m, $\rho_{Omin}$ = 50 m, $\left|V_{Ob}\right|=\left|V_{UAV}\right|=15$ m/s. $t_{n}$—the moment when the distance was the shortest; $t_{n-1}$—the moment 1 s before $t_{n}$; $t_{n+1}$—the moment 1 s after $t_{n}$.

**Figure 13:**
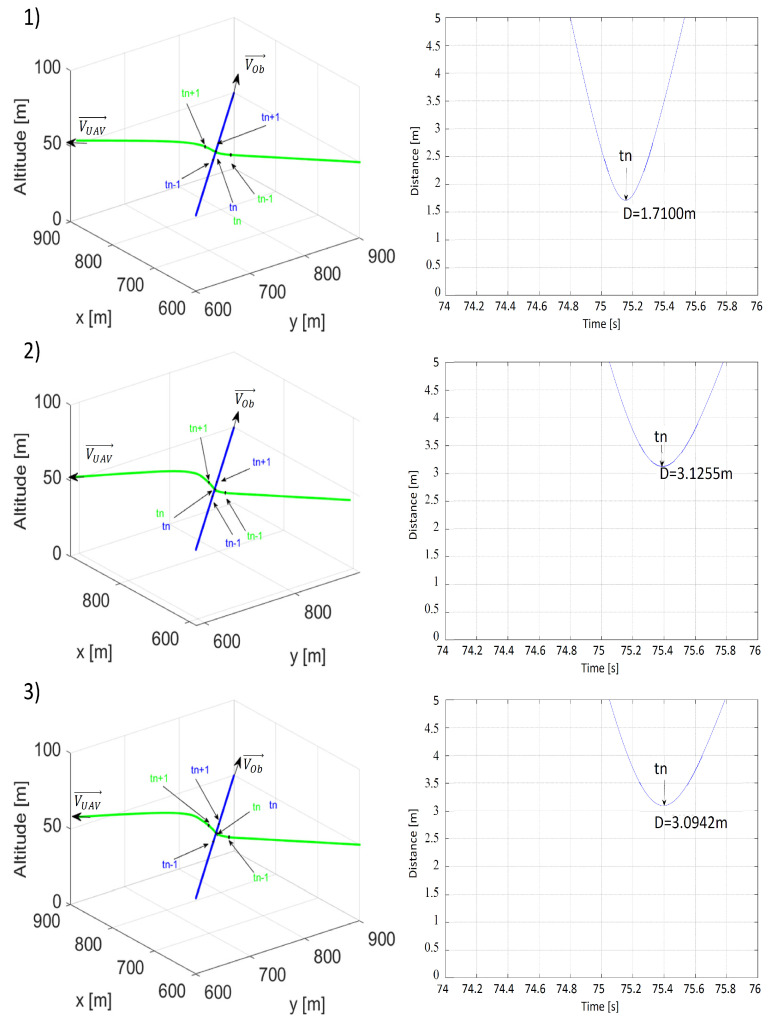
Time plots of paths (the UAV—green; the obstacle—blue) and distances between the UAV and the obstacle for (**1**) $\rho_{Lmin}$ = 30 m, $\rho_{Omin}$ = 25 m, $\left|V_{Ob}\right|=\left|V_{UAV}\right|=15$ m/s, (**2**) $\rho_{Lmin}$ = 30 m, $\rho_{Omin}$ = 50 m, $\left|V_{Ob}\right|=\left|V_{UAV}\right|=15$ m/s, (**3**) $\rho_{Lmin}$ = 30 m, $\rho_{Omin}$ = 75 m, $\left|V_{Ob}\right|=\left|V_{UAV}\right|=15$ m/s. $t_{n}$—the moment when the distance was the shortest; $t_{n-1}$—the moment 1 s before $t_{n}$; $t_{n+1}$—the moment 1 s after $t_{n}$.

Results of the last scenario simulating the danger of a perpendicular collision are presented in Figure 12 and Figure 13. Because, in this scenario, the collision point was the point at which the paths intersectdc (Figure 5), initial positions and speeds needed to be properly selected and unchangeable. Thus, similar to the previously described scenarios, the only changes occurred to distances $\rho_{Lmin}$ and $\rho_{Omin}$.

Both distances $\rho_{Lmin}$ and $\rho_{Omin}$ increased the minimal distance between the UAV and the obstacle, but these differences were not as crucial as in previous scenarios. When $\rho_{Lmin}$ = 10 m and $\rho_{Omin}$ = 25 m, the value of the minimal distance between the UAV and the obstacle became critical and made the collision risk unacceptable. Higher $\rho_{Lmin}$ and $\rho_{Omin}$ strengthened the effect of repulsion, which can be seen in Figure 12(2,3) and Figure 13(2,3) with the arcs of the UAV’s flight path becoming longer. In conclusion, to ensure that a safe avoidance distance was observed in situations carrying a threat of a perpendicular collision as well as for better overall results, $\rho_{Lmin}$ and $\rho_{Omin}$ had to not be lower than 30 and 50 m, respectively.

Table 1, Table 2 and Table 3 present the results of research into a new repulsive potential field approach displayed as minimal distance values seen in obstacle avoidance maneuvers for different combinations of $\rho_{Lmin}$, $\rho_{Omin}$ and $\left|V_{Ob}\right|$ in three collisions scenarios.

In the first scenario, where the UAV and the obstacle were moving directly toward one another, the minimum safe distance to the obstacle $\rho_{Omin}$ was of crucial significance to the value of the shortest distance, while the role of $\rho_{Lmin}$ was insignificant. This was true because the avoidance of a frontal collision requires that the distance between two moving objects is sufficient to achieve a safe maneuver. An increase in the obstacle’s speed decreased the distance needed for collision avoidance.

In the second scenario, where the UAV was flying behind the obstacle but was moving at a greater speed than it, the impact of both $\rho_{Omin}$ and $\rho_{Lmin}$ on the minimum avoidance distance was similar to that seen in the first scenario, with the exception being situations where the value of $\rho_{Lmin}$ was less than 20 m; then, the minimal collision avoidance distance decreased. It went back up when the relative speed between the UAV and the obstacle decreased, i.e., the obstacle speed grew from 0 to 10 m/s. Interestingly, the minimal collision avoidance distance decreased when the obstacle’s speed equaled the UAV’s speed. This could be explained by the fact that the value of the maximum of the potential function (15) was smallest when $V_{R}$ = 0.

In the final scenario, one where the UAV’s flight path was perpendicular to that of the obstacle, the smallest values of collision avoidance distance were obtained only for greater values of $\rho_{Lmin}$. An increase in $\rho_{Omin}$ had no significance and any value of this parameter higher than 50 m slightly decreased the strength of repulsion for the same values of $\rho_{Lmin}$.

To summarize, $\rho_{Omin}$ and $V_{R}$ play important roles in situations with a threat of frontal collisions, while $\rho_{Lmin}$ are of significance with respect to those presenting a danger of lateral collisions. In contrast to traditional spherical repulsive artificial fields, the multidimensional approach described above can separately adjust the strength of repulsive forces in situations concerning threats of both frontal and lateral collision. Of course, the range of the repulsive field should be greater in cases where there is a risk of a frontal collision which the proposed solution achieves as reflected in the results of carried-out simulations. This flexibility in designing repulsive fields around moving obstacles is the method’s main advantage. It allows adjustments with consideration for the maneuverability of nonholonomic vehicles as well as for maximum relative speeds between the UAV and the obstacle with the main principle being its ability to repulse the UAV from the obstacle’s line of momentary movement direction rather than from the obstacle itself, making it more suitable for dynamic environments.

## 5. Conclusions

The novel repulsive potential function described above can be deemed to be multidimensional because it is a function of the distance to the line of the obstacle’s velocity vector (the line of the momentary direction of movement), of the distance to the obstacle along this line, and of relative speed. As proven by the results of the present work’s simulations, it determined adjustments of the area of the repulsive force around the obstacle independently for preventing frontal and lateral collisions possible. The repulsive force acted perpendicularly to the line of the obstacle’s velocity vector and its strength decreased as the distance to the line and the obstacle increased. This made it well suited to nonholonomic UAVs because, in situations when such a vehicle comes closer to the obstacle, the repulsive force, even though it is still weak at that moment, decelerates the UAV and changes its direction to one that is perpendicular to the obstacle’s velocity vector. The main parameters that determine the minimal distance of collision avoidance are the distance to the line of the obstacle’s velocity vector (a key parameter in lateral collisions) and the distance to the obstacle (a key parameter in frontal collisions). In intermediate cases, carrying the risk of a collision, both play a role. The impact of relative speed on minimal avoidance distance was smaller in cases involving the danger of a frontal collision because, despite its higher values and an increase in the repulsive effect, it was canceled out by the inertia of the UAV’s dynamics. Simulation results proved that the novel repulsive potential function presented above was also effective in situations involving stationary obstacles as can be seen in Figure 11. An interesting result was seen in a simulation carrying a threat of a rear collision when relative velocity $V_{R}$ was equal to zero, where the obstacle avoidance distance was smaller than when $V_{R}$ was not zero Table 2. This can be explained using the equation describing the potential function (15), where the maximum value for $V_{R}$ = 0 was smaller resulting in less repulsion. However, for $V_{R}$ = 15 m/s ($V_{Ob}$ = 0 and $V_{UAV}$ = 15), the distance which the UAV had to avoid the collision was also smaller. This was caused by the vehicle’s inertia and even its correct response to the increased force of repulsion caused the UAV to penetrate deeper into the repulsive field area before it achieved setpoints imposed by the repulsive field. In every scenario simulating the risk of a frontal, perpendicular, and rear collision, the UAV was able to avoid obstacles at a minimum distance of 19 cm. Of course, this value cannot be accepted as a safe distance and it must be increased to ensure collision avoidance. With the UAV speed being approximately 15 m/s, minimal distances $\rho_{Omin}$ and $\rho_{Lmin}$ should not be lower than 50 and 30 m, respectively. Values for these parameters in the repulsive function should be adjusted to account for the UAV’s minimal turning radius, a result of its nonholonomic constraints. Further research, to be completed later, is needed to determine a function between values $\rho_{Omin}$, $\rho_{Lmin}$, and relative speed $V_{R}$ to ensure safe obstacle avoidance at any value of speed of both the UAV and the obstacle. To summarize, the proposed novel multidimensional potential function and the field of repulsive forces at the front of and behind the obstacle designed on its basis can be applied to nonholonomic UAVs in dynamic environments to achieve safe collision avoidance. 

## Figures and Tables

**Table 1:** Shortest recorded distances between the UAV and the obstacle obtained in simulations of the first collision avoidance scenario from Figure 6, Figure 7 and Figure 8.

$\left|V_{\mathrm{Ob}}\right|$ = 10(1); 15(2); 30(3), $\left|V_{\mathrm{UAV}}\right|$ = 15	$\rho_{\mathrm{Lmin}}$ = 10 m	$\rho_{\mathrm{Lmin}}$ = 20 m	$\rho_{\mathrm{Lmin}}$ = 30 m
$\rho_{Omin}$ = 25 m	-	1.0478 m(2)	-
$\rho_{Omin}$ = 50 m	4.9825 m(2)	5.7354 m(1) 4.0886 m(2) 3.0532 m(3)	4.0805 m(2)
$\rho_{Lmin}$ = 75 m	-	8.8784 m(2)	-

**Table 2:** Shortest recorded distances between the UAV and the obstacle obtained in simulations of the second collision avoidance scenario from Figure 9, Figure 10 and Figure 11.

$\left|V_{\mathrm{Ob}}\right|$ = 0 (1); 10(2); 15(3), $\left|V_{\mathrm{UAV}}\right|$ = 15	$\rho_{\mathrm{Lmin}}$ = 10 m	$\rho_{Lmin}$ = 20 m	$\rho_{\mathrm{Lmin}}$ = 30 m
$\rho_{Omin}$ = 25 m	-	14.3481 m(2)	-
$\rho_{Omin}$ = 50 m	28.1526 m(2)	11.444 m(1) 41.383 m(2) 34.811 m(3)	41.3553 m(2)
$\rho_{Lmin}$ = 75 m	-	50.3645 m(2)	-

**Table 3:** Shortest recorded distances between the UAV and the obstacle obtained in simulations of the third collision avoidance scenario from Figure 12 and Figure 13.

$\left|V_{\mathrm{Ob}}\right|$ = 15, $\left|V_{\mathrm{UAV}}\right|$ = 15	$\rho_{\mathrm{Lmin}}$ = 10 m	$\rho_{\mathrm{Lmin}}$ = 20 m	$\rho_{\mathrm{Lmin}}$ = 30 m
$\rho_{Omin}$ = 25 m	-	-	1.7100 m
$\rho_{Omin}$ = 50 m	0.1905 m	0.8759 m	3.1255 m
$\rho_{Lmin}$ = 75 m	-	-	3.0942 m

## Data Availability

Not applicable.
